# Supplementary material for: IL-8 Secreted by Gastric Epithelial Cells Infected with Helicobacter pylori CagA Positive Strains Is a Chemoattractant for Epstein–Barr Virus Infected B Lymphocytes
Source: Viruses. 2023 Feb 28;15(3):651. doi: 10.3390/v15030651 (PMC10054738; doi:10.3390/v15030651)
Supplement: Supplementary file 1 [file viruses-15-00651-s001.zip › viruses-2160778-supplementary.pdf]

Article

IL-8 Secreted by Gastric Epithelial Cells Infected with Helicobacter pylori CagA Positive Strains is a Chemoattractant for Epstein-Barr Virus Infected B Lymphocytes

Diana A. Domínguez-Martínez [1], José I. Fontes-Lemus [1], Alejandro García-Regalado [1], Ángel Juárez-Flores [1,2] and Ezequiel M. Fuentes-Pananá [1,*]

[1] Research Unit on Virology and Cancer, Children's Hospital of Mexico Federico Gómez, Mexico City 06720, Mexico; alheli43@hotmail.com (D.A.D.-M.); nachopnc.15@gmail.com (J.I.F.-L.); alegregalado@yahoo.com.mx (A.G.-R.); angel.juarez.bqd@gmail.com (Á.J.-F.)

[2] Unidad de Investigación Médica en Inmunoquímica, Hospital de Especialidades, Centro Médico Nacional Siglo XXI, Instituto Mexicano del Seguro Social, Ciudad de México, 06720, Mexico

* Correspondence: empanana@yahoo.com or Ezequiel.Fuentes@alumni.bcm.edu; Tel.: +52-55-4434-9663

Supplementary Table S1. Genes of the signature of chemokine and chemokine receptor used for bioinformatic analysis.

**Gene**		**References**
**Ligand**	**Receptor(s)**	
CXCL8 (IL-8)	*CXCR1, CXCR2*	[16,38-43,47]
CXCL9	*CXCR3*	[44]
CXCL10	*CXCR3*	[41,44-45,47]
CXCL11	*CXCR3*	[44,47]
CXCL12	*CXCR4, CXCR7*	[44,46-47]
CXCL13	*CXCR5*	[45,49-51]
CCL19	*CCR7*	[48]
CCL20	*CCR6*	[42-43,45,48,52]
CCL21	*CCR7*	[48,53]
CCL25	*CCR9*	[47,54]

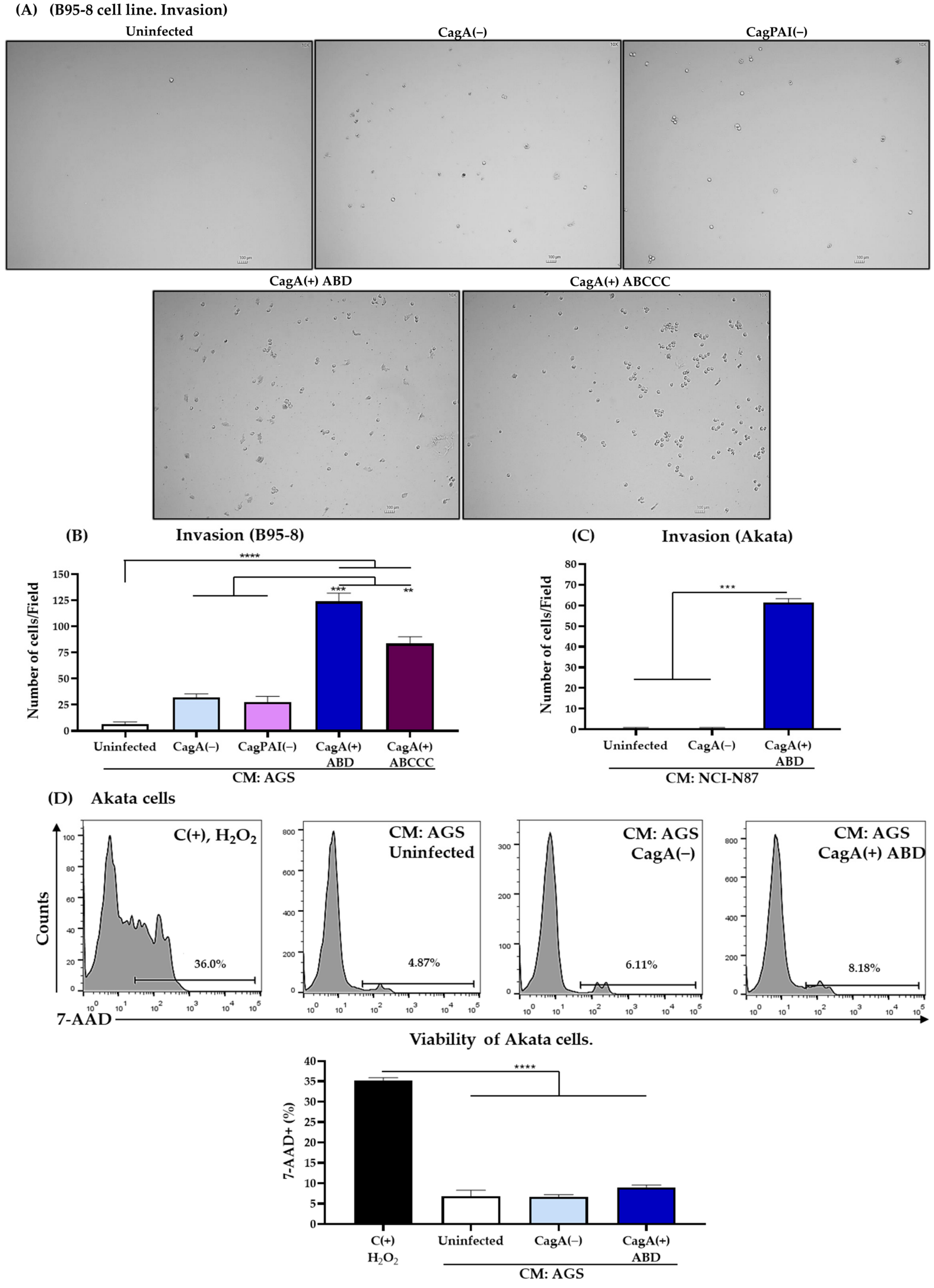

Supplementary Figure S1. EBV-infected B lymphocytes are chemoattracted by components of conditioned media (CM) obtained from gastric epithelial cells infected with *H. pylori* strains. (**A**) Representative images of B95-8 cell line (EBV-positive cells, latency III) chemoattracted to CM of AGS cells uninfected or infected with *H. pylori* CagA(−) or CagA(+) strains. The scale bars indicate 100 μm, magnification 10×. (**B**) Plot of the mean numbers of chemoattracted B95-8 cells. (**C**) Plot of chemoattracted Akata cells to CM from

NCI-N87 cells infected with *H. pylori* strains. Graphs represent the mean ± SEM of the number of chemoattracted cells counted from 5 random microscope fields of three independent experiments.(**D)** Flow cytometry analysis to evaluate viability of Akata cells cultured with CM obtained from AGS cells uninfected or infected with *H. pylori* CagA(−) and CagA(+) ABD strains for 48 h (time that lasted the chemoattraction assays). The positive cells to 7-AAD (percentage of dead cells) are indicated in the histogram. Hydrogen peroxide (H_2O_2) was used as positive control (C+) of dead cells. Graph represents the mean ± SEM of the percentage of positive cells to 7-AAD of three independent experiments. A non-parametric Kruskal–Wallis and Dunn´s post-hoc tests were used for the statistical analysis, ** $p < 0.01$, *** $p < 0.001$, **** $p < 0.0001$.

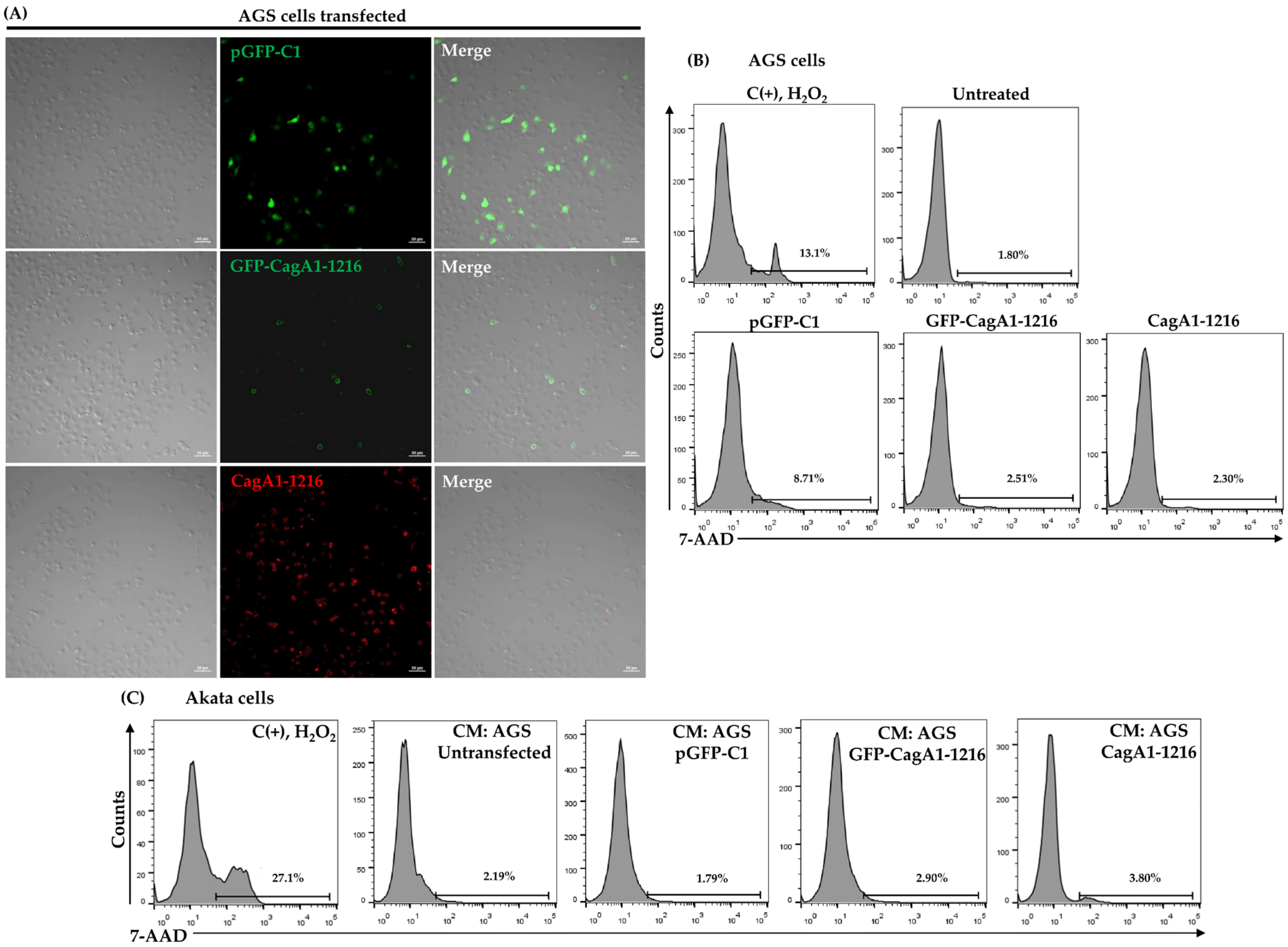

Supplementary Figure S2. Overexpression of CagA in AGS cells. (**A**) Immunofluorescence of AGS cells transfected with the pGFP-C1 (empty vector), and full-length CagAs (GFP-CagA1-1216 and CagA1-1216) plasmids. The scale bars indicate 50 µm, magnification 20x. Flow cytometry analysis to evaluate viability of transfected AGS cells after harvesting the conditioned media (**B**), and of Akata cells maintained in the conditioned media (CM) of transfected AGS cells for 48 h (the time that the chemoattraction assays lasted) (**C**). (**B**,**C**) Viability assays were performed twice, and one is shown as an example. The positive cells to 7-AAD (percentage of dead cells) are indicated in each histogram. Hydrogen peroxide (H_2O_2) was used as positive control (C+) of dead cells.

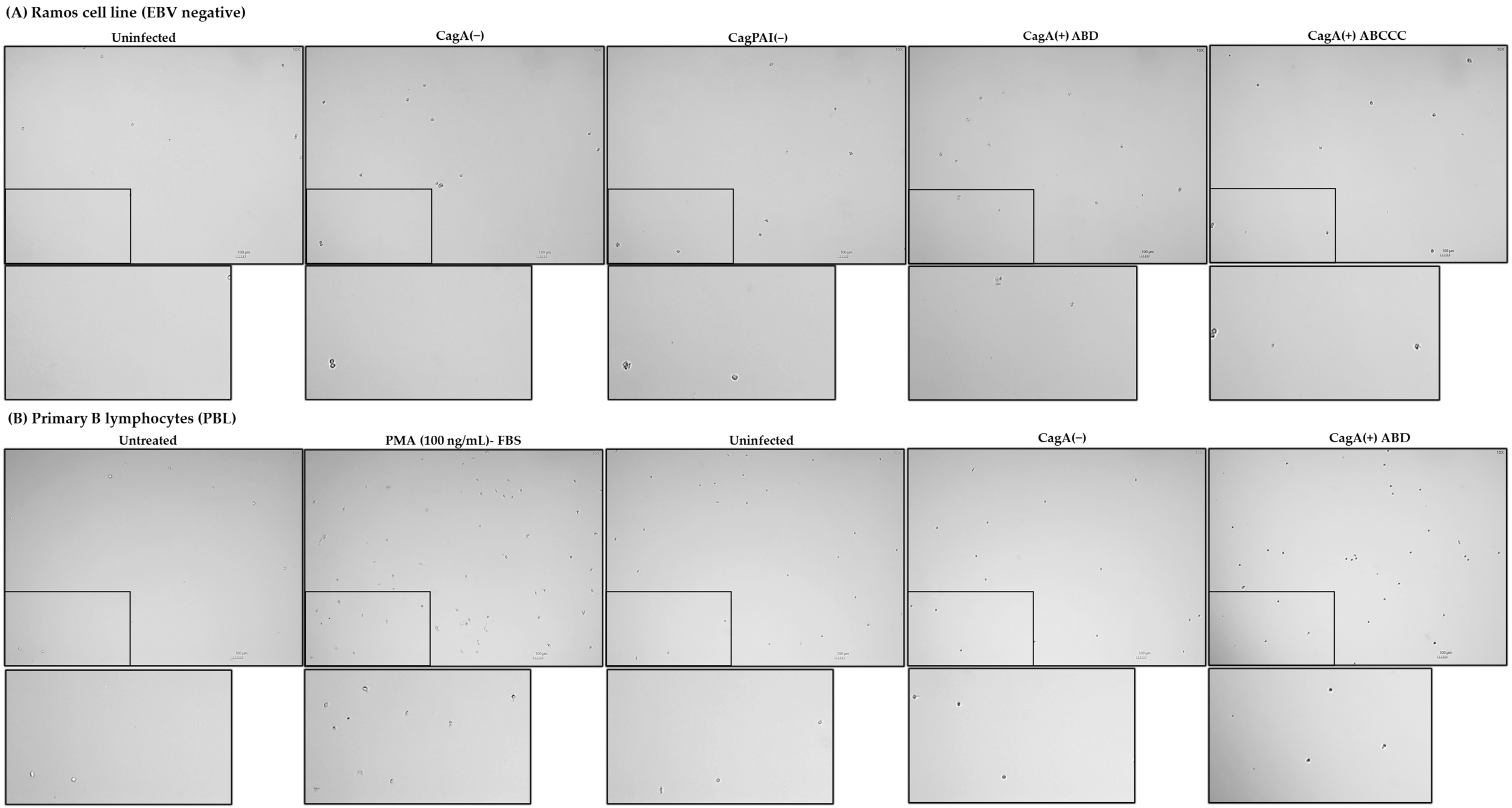
(A) Ramos cell line (EBV negative)
Uninfected
CagA(–)
CagPAI(–)
CagA(+) ABD
CagA(+) ABCCC
(B) Primary B lymphocytes (PBL)
Untreated
PMA (100 ng/mL)- FBS
Uninfected
CagA(–)
CagA(+) ABD

Supplementary Figure S3. An active *H. pylori* infection is needed to chemoattract EBV-infected B lymphocytes. Representative images of migration assays of the Ramos cell line (**A**) and Primary B lymphocytes (PBLs) (**B**) to conditioned media (CM) from AGS cells infected with *H. pylori* CagA(−) or CagA(+) strains (the scale bars indicate 100 µm), magnification 10x. PBLs treated with PMA were used as positive control for invasion.

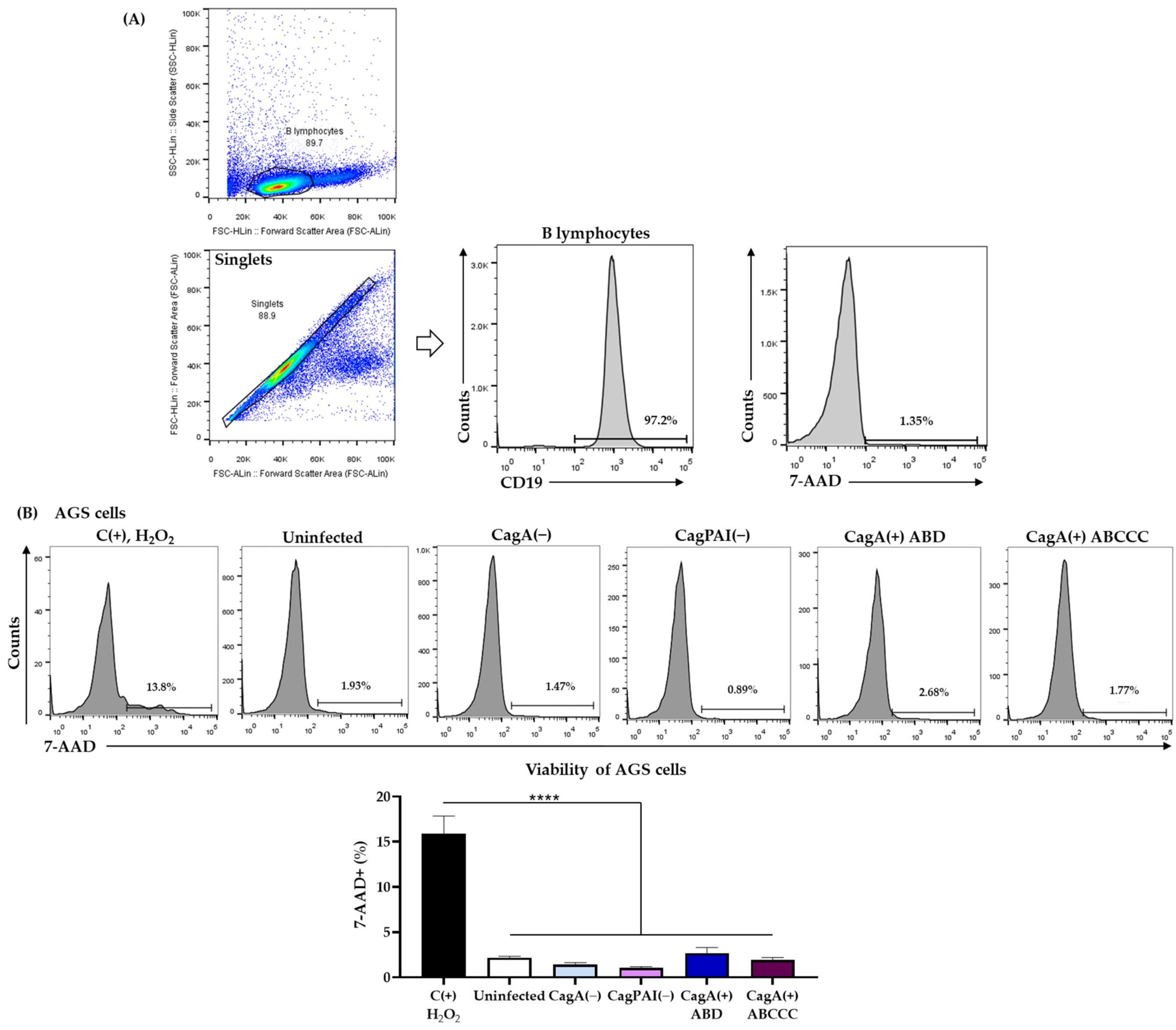

Supplementary Figure S4. (**A**) An example of the primary B lymphocytes (PBLs) purification strategy carried out from three healthy donors. Side scatter and forward scatter plots were used to select the lymphocyte gate, doublets were then excluded and B lymphocyte purity was assessed by CD19 expression and viability with 7-AAD. **(B)** Flow cytometry analysis to evaluate viability of AGS cells infected with *H. pylori* strains at the time that conditioned media (CM) were harvested. The positive cells to 7-AAD (percentage of dead cells) are indicated in each histogram. Hydrogen peroxide (H_2O_2) was used as positive control (C+) of dead cells. Graph represent the mean ± SEM of the percentage of positive cells to 7-AAD of three independent experiments. A non-parametric Kruskal–Wallis and Dunn´s post-hoc tests were used for the statistical analysis, **** $p < 0.0001$.

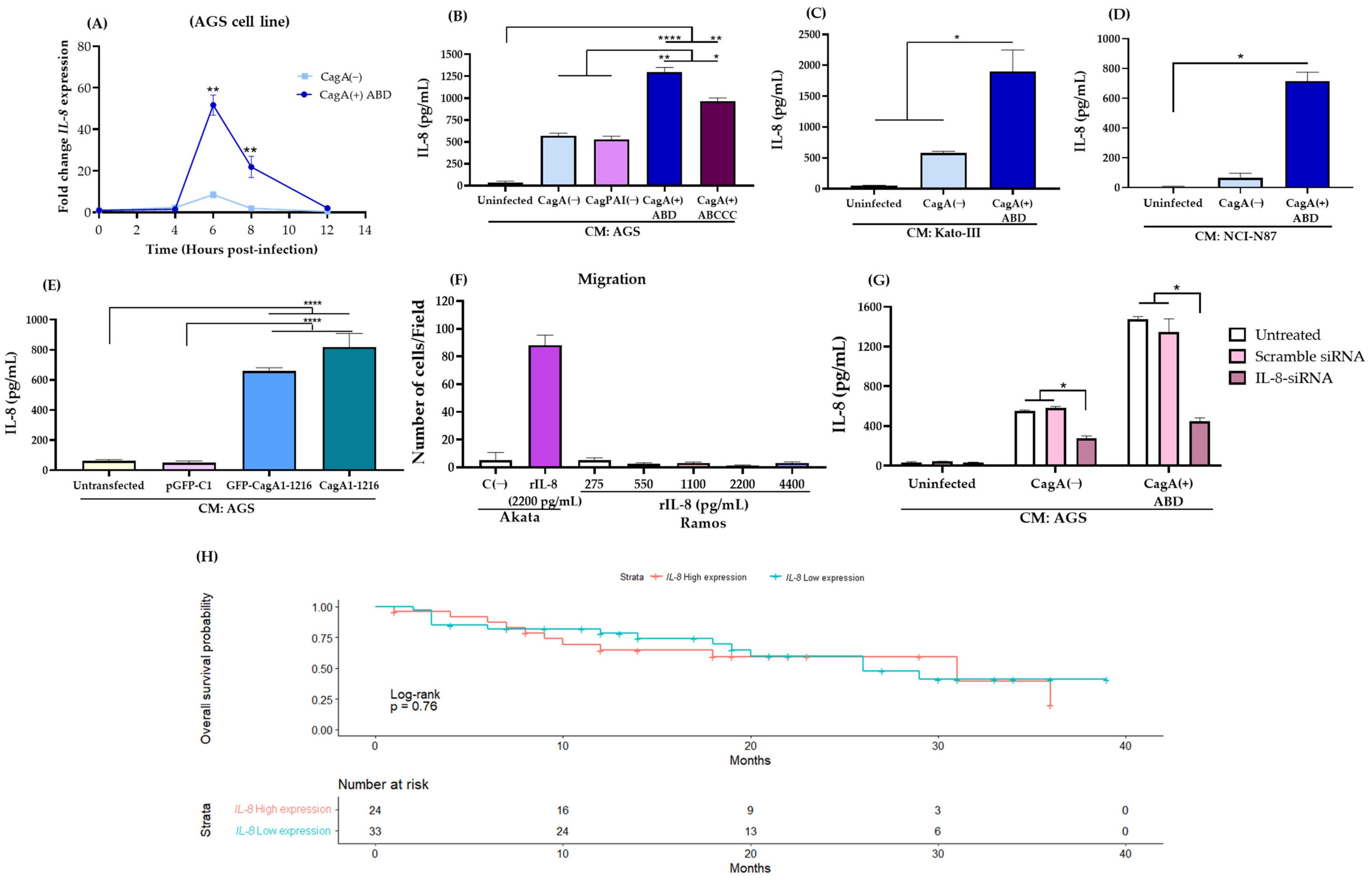

(A)
(AGS cell line)
Fold change IL-8 expression
Time (Hours post-infection)
CagA(−)
CagA(+) ABD
(B)
IL-8 (pg/mL)
Uninfected
CagA(−)
CagPAI(−)
CagA(+) ABD
CagA(+) ABCCC
CM: AGS
(C)
Uninfected
CagA(−)
CagA(+) ABD
CM: Kato-III
(D)
Uninfected
CagA(−)
CagA(+) ABD
CM: NCI-N87
(E)
Untransfected
pGFP-C1
GFP-CagA1-1216
CagA1-1216
CM: AGS
(F)
Migration
Number of cells/Field
C(−)
rIL-8 (2200 pg/mL)
Akata
275
550
1100
2200
4400
rIL-8 (pg/mL)
Ramos
(G)
Untreated
Scramble siRNA
IL-8-siRNA
Uninfected
CagA(−)
CagA(+) ABD
CM: AGS
(H)
Strata
IL-8 High expression
IL-8 Low expression
Overall survival probability
Log-rank
p = 0.76
Months
Number at risk
Strata
IL-8 High expression 24 16 9 3 0
IL-8 Low expression 33 24 13 6 0

Supplementary Figure S5. IL-8 is responsible for the chemoattraction of EBV-infected B lymphocytes. **(A)** Fold-change gene expression of *IL-8* in AGS cells infected with *H. pylori* CagA(−) or CagA(+) ABD strains at different time points of infection. (**B-D**) IL-8 concentration in conditioned media (CM) from AGS, Kato-III and NCI-N87 cell lines uninfected or infected with *H. pylori* strains. (**E**) IL-8 concentration in conditioned media (CM) from AGS cells untransfected or transfected with the empty vector (pGFP-C1), or the full-length CagAs (GFP-CagA1-1216 and CagA1-1216) at 28 h post-transfection. (**F**) Plot of chemoattraction assays of Akata and Ramos cell lines towards different rIL-8 concentrations. (**G**) IL-8 concentration in conditioned media (CM) from AGS cells untreated or transfected with a scramble siRNA-A or a specific human *IL-8* siRNA (h*IL-8* siRNA) and further infected with *H. pylori* CagA(−) or CagA(+) strains. (**H**) Kapplan-Meyer survival analysis based on *IL-8* expression in the database of the TCGA consortium [5]. (**A,E,G**) A 2-way ANOVA for multiple comparison and Tukey´s post-hoc test were used to identify statistical differences. (**B-D,F**) A non-parametric Kruskal–Wallis and Dunn´s post-hoc tests were used for the statistical analyses. * $p < 0.05$, ** $p < 0.01$, **** $p < 0.0001$.

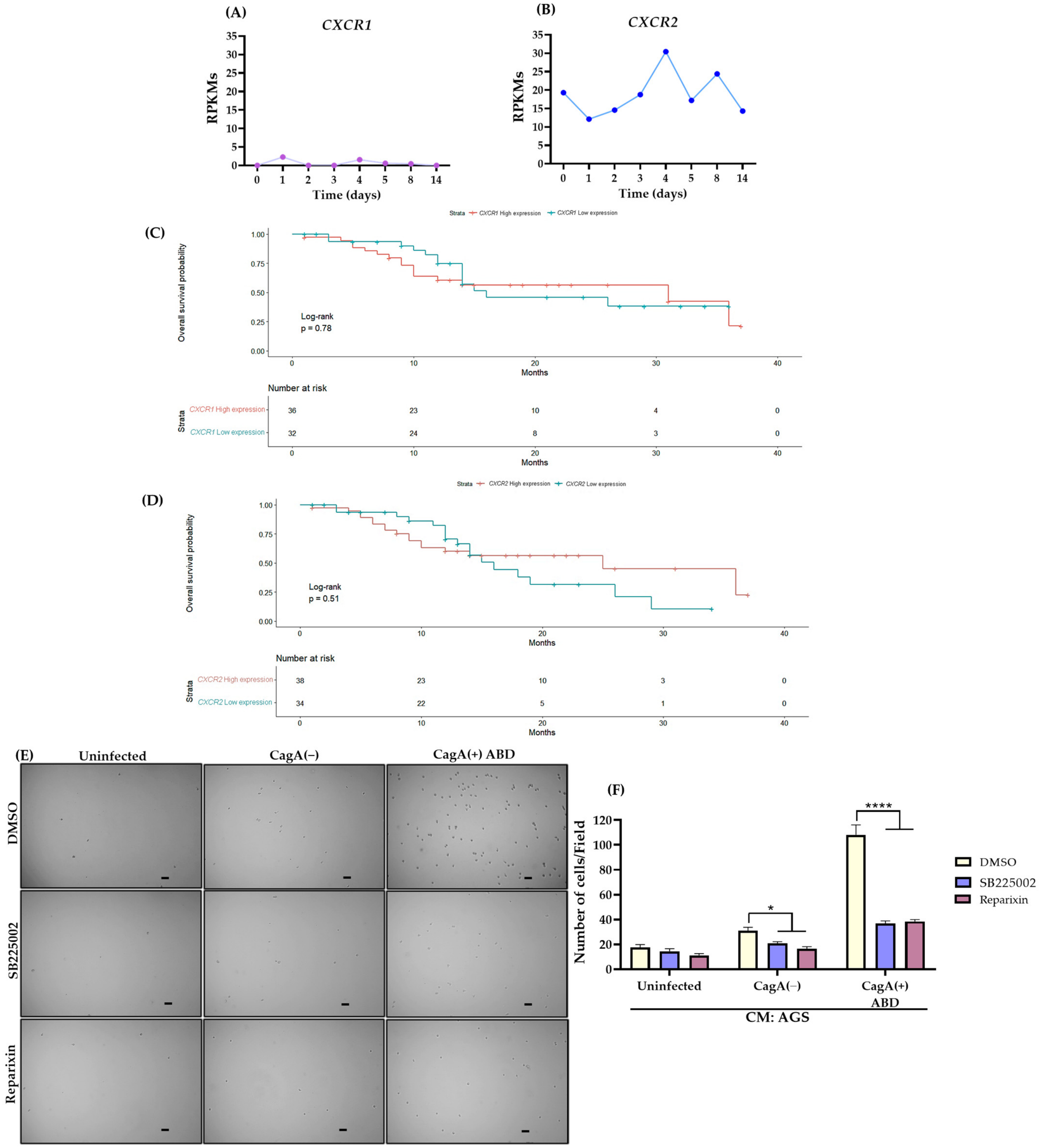

Supplementary Figure S6. IL-8 promotes activation of ERK1/2 and p38 MAPK through CXCR2. **(A,B)** Transcriptomic analysis of *CXCR1* and *CXCR2* in naïve B lymphocytes infected with EBV [64]. Graphs show Reads Per Kilobase Million (RPKMs) of *CXCR1* and *CXCR2* during the first two weeks after EBV infection. (**C-D**) Kapplan-Meyer survival analysis based on *CXCR1* and *CXCR2* expression of the gastric cancer database of the TCGA consortium [5]. (**E,F**) Invasion assays of Akata cells pretreated with inhibitors of CXCR2 and chemoattracted to conditioned media (CM) from AGS cells infected with *H. pylori* CagA(−) or CagA(+) ABD strains. (**E**) Representative images of Akata cells chemoattracted to CM from AGS cells. The scale bars indicate 100 μm, magnification 10×. (**F**) Graph represent the mean ± SEM of the number of invading cells counted from 5 random microscope fields of three independent experiments. A 2-way ANOVA for multiple comparison and Tukey´s post-hoc test were used to identify statistical differences, * $p < 0.05$, and **** $p < 0.0001$.

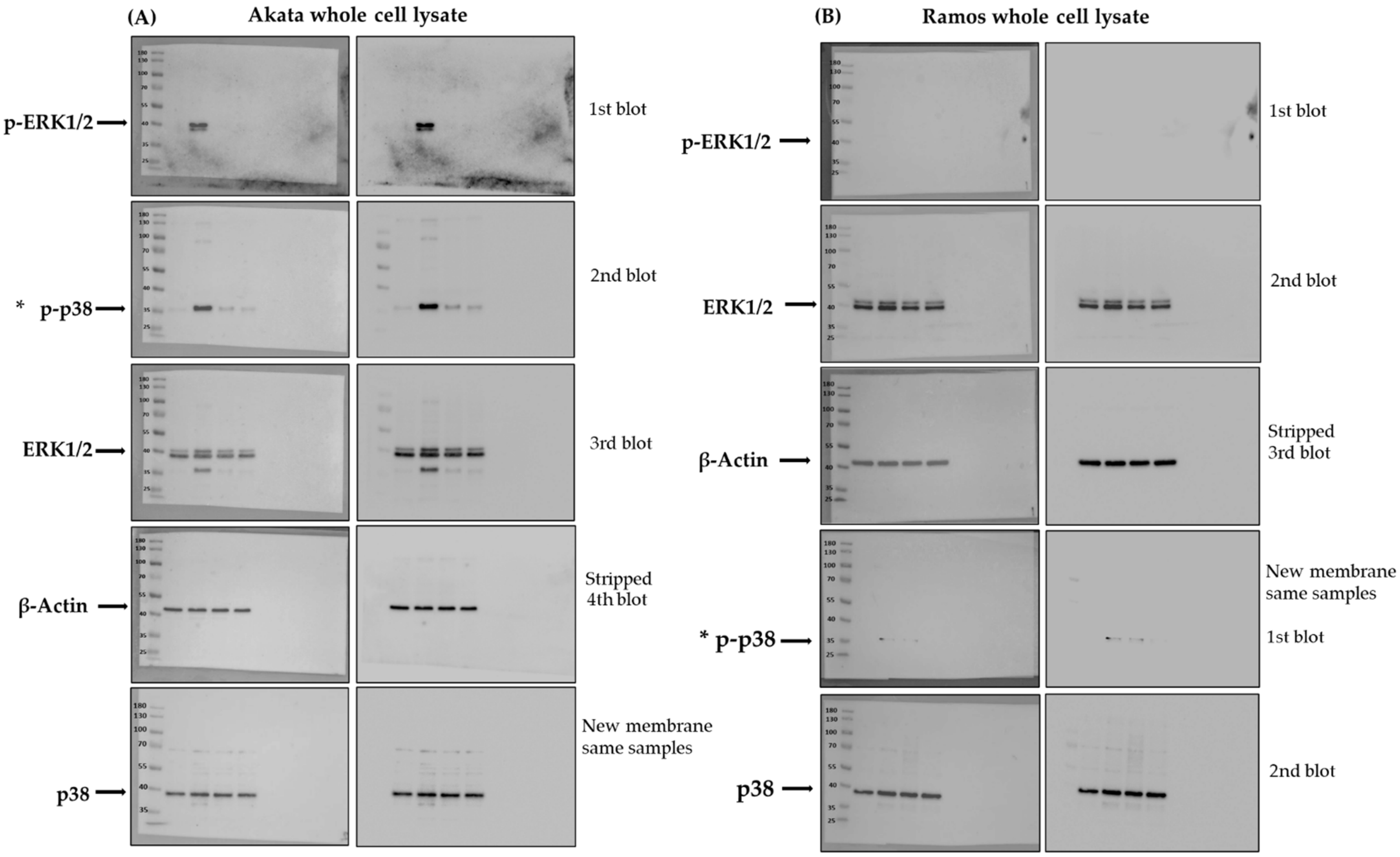

Supplementary Figure S7. Western blot analysis of whole cell lysates of Akata **(A)** and Ramos **(B)** cell lines. Uncropped full-length pictures of Western blot membranes presented in **Figure 5**D,E. Membranes were blotted with multiple antibodies as are indicated in the image. The blots were homogenized in brightness and contrast to improve their presentation. The arrows indicate the band of interest, and the asterisk the migration of the phosphorylated (p) protein.
